# School-Based Sexual and Reproductive Health Services for Prevention of Adolescent Pregnancy in the Hoima District, Uganda: Cluster Randomized Controlled Trial

**DOI:** 10.3390/mps2010021

**Published:** 2019-03-04

**Authors:** Gloria Kirungi Kasozi, Julius Kasozi, Frank Pio Kiyingi, Miph Musoke

**Affiliations:** 1 School of Post graduate Studies, Nkumba University, Entebbe 237, Uganda; kfrankpio@yahoo.com (F.P.K.); ar@nkumbauniversity.ac.ug (M.M.); 2 Department of Public Health, School of Medicine, Uganda Christian University, Mukono 4, Uganda; 3 Public Health Office, United Nation High Commissioner for Refugee, Kampala 3813, Uganda; jmkasozi@gmail.com

**Keywords:** sexual and reproductive health, adolescent, pregnancy, school-based, intervention, utilization, prevention, information

## Abstract

Uganda has persistently had high adolescent pregnancy prevalence; 25% for the last 10 years. This protocol presents the design of a Cluster Randomized Controlled Trial (CRCT) to investigate the effectiveness of School-Based Sexual and Reproductive Health (SBSRH) interventions on prevention of pregnancy among school girls aged 15–19 years in the Hoima District, Uganda. 18 secondary schools (clusters) will be selected using cluster sampling and allocated 1:1 into control or intervention group stratified by geographical location. 1080 (60 each cluster) participants/girls aged 15–19 years will be selected using simple random sampling. The intervention group will receive tailored SRH information, in-school medical care and referral over 12 months. The control group will receive no intervention from the research team; however, they can access alternative services elsewhere if they wish. Data will be obtained at baseline, 6 months and 12 months. The outcomes are reduction in occurrence of pregnancy, utilization of SRH services and sexual behavioral change. To our knowledge, this is the first CRCT providing combined SRH interventions for prevention of adolescent pregnancy in Uganda. If effective, it could have great potential in preventing adolescent pregnancy. Trial Registration: Pan African Clinical Trial Registry (PACTR201810882140200) Registered on 16 October 2018.

## 1. Introduction

Adolescence is a transitional phase of growth and development between childhood and adulthood, mainly referring to individuals between the age of 10 and 19 years [1]. The United Nations Children’s Fund (UNICEF) defines an adolescent pregnancy as a young girl aged 13–19 years who becomes pregnant or has had a live birth [2]. Half of the world’s population is under 25 years old and the population of young people aged between 10–25 years is estimated to be 1.8 billion with about 85% living in the developing world [2]. 49% of girls in least developed countries (LDCs) marry before they turn 18, while 10%–40% of young unmarried girls have had an unintended pregnancy, giving rise to about 14 million children worldwide who are born every year to young married and unmarried women aged 15 to 19 years [2]. In addition, the United Nation Population Fund estimates that every day in developing countries, 20,000 girls under age 18 give birth resulting into 7.3 million adolescent births a year [3].

Uganda has consistently had high rates of adolescent pregnancy at 25% for the last ten years. These rates have been coming down gradually but steadily: in 1995, it was 43%, in 2000–2001 it dropped to 31% [4] mainly because of the sexuality education program that started in schools and communities targeting young girls.

Early marriage, early initiation of sex and lack of access to reproductive health information and services are the leading drivers of adolescent pregnancy in the world [2,5,6]. A study conducted in 2018 on factors associated with teenage pregnancies among school going teenagers in eastern Uganda showed inadequate information about reproductive health with 75% of the respondents believing that the minimum age for conception is 14 years. In addition, bad peer groups, enticement with gifts and poverty were identified as the most common causes of teenage pregnancy [6]. A recent study conducted among the very young adolescents in South Western Uganda revealed that 7.6% had ever had sex yet 90% of the adolescents were not using any form of protection from HIV and pregnancy [7]. It has also been documented that the majority of adolescents usually consult their mothers and aunts for Sexual and Reproductive Health (SRH) information [8], which poses a gap in accuracy of information that is provided by the immediate relatives.

However, adolescent pregnancies may occur as a result of other factors: customs and traditions that lead to early marriage, adolescent sexual behavior, which may also be influenced by alcohol and drugs, lack of education and information about reproductive sexual health, lack of access to tools that prevent pregnancies, peer pressure to engage in sexual activity, incorrect use of contraception, sexual abuse that leads to rape, poverty, exposure to abuse, violence, family strife at home, low self-esteem and low educational ambitions or goals [9,10]. It is also important to note that adolescent pregnancy is not always a result of a deliberate choice; often, unintended pregnancy among adolescent girls is a consequence of little or no access to Adolescent Sexual and Reproductive Health (ASRH) information and services [3]. The majority of adolescent girls who become pregnant are married and/or pressured to have a child, while for others, pregnancy often results from abusive, forced or coerced sex [11]. The limited awareness on consequences of adolescent pregnancy, inaccessibility to ASRH services and social protection of the adolescent girls within the community increases the risk of becoming pregnant [3].

In sub-Saharan Africa, adolescent births are more likely to occur among poor, less educated and rural populations because of reasons including lack of access to contraception, early marriage, poor or limited access to education, cultural norms and poverty, to mention but a few [12,13]. An adolescent pregnancy poses a challenge to maternal and child survival, because pregnant adolescents and adolescent mothers are likely to be uneducated, unemployed and poor and might not seek or utilize health care services for either themselves or their newborns’ at critical times [14,15]. Thus, increased risks of maternal morbidity and mortality have been noted among pregnant and adolescents mothers [16]. This means that efforts to reduce maternal and child mortality need to focus on adolescents girls too.

Adolescent pregnancy and motherhood has detrimental socioeconomic and psychological outcomes for the young mother, her child, and her young siblings. When a girl becomes pregnant, her education may end, her job prospects diminish, she becomes more vulnerable to poverty, her health often suffers and the complications from pregnancy and childbirth are the leading cause of death among adolescent girls. The child on the other hand is likely to live in poverty, grow up without a father, become a victim of neglect or abuse, do less well at school, become involved in crime, abuse drugs and alcohol, eventually become a teenage parent themself and begin the cycle all over again [2,3].

The consequences of adolescent pregnancy include premature births, low birth weight of the newborn, high risks for medical complications such as obstructed labor, obstetric fistula, leakage of urine and/or feces and mortality [9]. Adolescent girls account for 14% of the estimated 20 million unsafe abortions performed each year, which result in approximately 68,000 deaths. This is an alarming public health problem globally and requires urgent multi-sectoral attention [9].

In 2013, there were nearly 300,000 abortions in Uganda, which is equivalent to 54 abortions per 1000 women of reproductive age, or one abortion for every 19 such women. This rate is far higher than the average rate for Eastern Africa, which is 36 abortions per 1000 women [17]. In addition, the Ministry of Health in Uganda estimated that abortions accounted for 26% of maternal mortality in 2008; this is higher than the 18% in the Eastern African region and the 13% in the world as a whole [1,17]. The Uganda Demographic Health Survey (UDHS), conducted in 2016, estimated the pregnancy-related mortality ratio to be at 368 death per 100,000 live births and adolescents 15–19 years contributing 17% of all pregnancy related mortality rate in Uganda. Additionally, 15 out of every 1000 Ugandan women of reproductive age were treated for abortion complications that year [4]. Therefore, it is paramount to pay special attention to adolescents to prevent maternal mortality due to unsafe abortions among the adolescents.

School nurses have long managed medical emergencies, helped students with chronic conditions, provided health education and immunizations and screened students’ vision and hearing; however, since the inception of these school clinics by nurses, heated debates have raged in communities across the country over whether they should provide contraceptives on-site, which clearly illustrates the role of society and resource persons in the health of the adolescents [18,19].

Several interventions have been proposed to reduce adolescent pregnancy; in particular, early education interventions have been linked to reduction in pregnancy and improvement in learning [20]. Additionally, in the absence of strong evidence that abstinence-only or abstinence-plus school-based programs affect sexual activity, the prohibition of contraceptive education in school-based pregnancy prevention programs prevents student exposure to information that has the greatest potential to decrease the pregnancy rate [21]. A recent study, conducted in Uganda on access and utilization of SRH services, found that the counseling on abstinence and other SRH issues is the most accessed and utilized service [8]. Qualitative data from a health project in Uganda shows that students can access these services while in transit to school without their parents’ knowledge and this was evidenced by the significant increment (10%) in contraceptive use among the 10–24 years during the school term compared to holiday period [22]. A study conducted in Uganda on perceptions of adolescents on SRH education by parents and other stakeholders revealed that peers and media were the main source of SRH information while mothers were perceived to provide more sexuality information to adolescents than fathers [23], this therefore indicates the need for regulation of media and the need to train more peer educators since they are the primary sources of information for adolescents.

A systematic review of 15 Randomized Controlled Trials (RCTs) conducted in developed countries on interventions for preventing unintended pregnancies among adolescents revealed that single (education) interventions were not found to be effective while combinations of interventions (educational and contraceptive access) to improve education and contraceptive access were found to reduce unintended pregnancies among adolescents. A combination of educational and contraceptive-promoting interventions appear to reduce unintended pregnancy among adolescents [24,25]. It is in this regard that this study seeks to implement a combination of education and ASRH services in a bid to reduce the occurrence of adolescent pregnancy among school girls aged 15–19 years. Key gaps that this study addressed include: (1) use of three tier interventions, i.e., education/behavioral change, services and referrals; (2) focus on school girls; (3) within an African context.

This study on the prevention of adolescent pregnancies among girls in secondary schools hinged on several theories aimed at changing behavior and primary prevention of adolescent pregnancy using school-based sexual and reproductive health services. An a priori approach will be used because the intervention proposed will be derived from existing theories without appealing to any individual experiences; the proposed model will then be implemented and tested during this research. The two main theories and approaches that will be used include: the Health Belief Model (HBM) and Trans-theoretical Model [26,27,28].

**Rationale:** This study is particularly important because it seeks to identify feasible and tested measures to promote safe sexual behavior intended to prevent adolescent pregnancy across the world. Uganda in particular has persistently had an unacceptably high adolescent pregnancy prevalence of 25% for the last 10 years compared to 16% globally [4]. This means that one in every four (25%) adolescent girls between 15 and 19 years in Uganda is pregnant or has had their first child. Despite the numerous studies that have been conducted worldwide and in Uganda on the use of school-based sexual and reproductive health services, school-based sexual and reproductive health programs are widely accepted as an approach to reducing high-risk sexual behavior among adolescents; however, these face numerous challenges in the implementation and scale-up because of the exposure of contraceptives to the school-going adolescents. In addition, incentive-based interventions that focus on keeping young people in secondary school generally reduce adolescent pregnancy; thus, there is a continued need to provide health services to adolescents using various approaches. Schools may be a good place in which to provide these services [29]. Therefore, this study focused in particular on the provision of comprehensive SRH package to adolescents in school for the reduction in risky behavior and prevention of pregnancy.

There is very limited evidence focusing on the provision of multiple interventions used in the prevention of adolescent pregnancy in both developing and developed countries. Therefore, this study therefore seeks to implement multiple interventions through a School-Based Sexual and Reproductive Health (SBSRH) model which will include both SRH information and services in selected secondary schools and will investigate the effects of providing SBSRH services on prevention of pregnancy among adolescent girls in school aged 15–19 years in the Hoima District, Uganda.

**Purpose of the study**: The purpose of the study is to determine the effect of providing School-Based Sexual and Reproductive Health information and services on the prevention of adolescent pregnancy among girls in school aged 15 to 19 years in the Hoima District, Uganda. The provision of SRH services on the school grounds was aimed at removing the barriers to access of these services among sexually active adolescents and youths so as to enable timely access to and utilization of SRH services. In addition, the study will seek to advance a suitable school-based SRH model for use in adolescent pregnancy prevention programs in Uganda and other developing countries.

**Significance of the study**: The results of the study will provide scientific evidence on the immediate, basic and underlying factors associated with adolescent pregnancies among school girls, barriers to access of SRH information and services as well as provide information on the burden of adolescent pregnancy among school girls.

Moreover, the study will provide evidence for the urgent need of combined interventions, which include SRH information, services and referral in schools. The sexual and reproductive health school model is intended to reduce barriers faced by adolescents in school in accessing accurate and timely SRH information and services in order to reduce unintended pregnancies; therefore, this study will benefit the adolescents and other stakeholders to improve access and availability of Adolescent SRH services.

The evidence from this study will be used to inform policy and planning mainly in Uganda and the African region on the effective evidence based interventions that have potential to reduce the adolescent pregnancy burden among school girls in the communities.

The study results will act as the basis for strengthening regulations and guidance on accessibility and availability of SRH services for the adolescent girls as well as provide statistics for advocacy, programming and policy development for integration of SRH services into the already existing school clinics. Implementation of the combined interventions will go a long way in contributing to the reduction of maternal and child morbidity as well as mortality by reducing unintended pregnancies among this vulnerable population.

**Aim of the study**: To develop a framework for the introduction of SRH services for adolescents in schools with the aim of improving adolescent SRH health by identifying barriers to services, surveying the current state of SRH among adolescents and assessing how adolescents respond to specific forms of SRH interventions.

## 2. Methodology

The study will use an experimental research design to assess the effect of the school-based SRH services on occurrence of the adolescent pregnancy among the school girls aged 15–19 years. The Cluster Randomized Controlled Trial (CRCT) design with qualitative and quantitative components will be used as a gold standard in conducting this impact evaluation research so as to maximize comparability between the intervention and control groups, and hence give a strong evidence of a causal relationship between the intervention and the outcome [30,31,32].

The unit of randomization will be the 18 clusters (school) and 1080 individuals (girls); therefore, the cluster (school) of the eligible girls will be randomly allocated to either the intervention (9 schools) or the control (9 schools) arms of the study.

Blinding will be done to prevent systematic biases stemming from foreknowledge of group allocations among the participants and research aim outcomes as per RCT study standards [31]. Pre-determined information, such as the research hypothesis, will be concealed from the participants, data collectors, teachers and data analysts so as to prevent biases at all levels of the research. The participants will not be made aware of the research hypothesis and will be assigned any group accordingly.

In order to avoid misleading biases, the study will use the Intent To Treat (ITT) approach for analysis. Every participant allocated to a treatment group is considered to be part of the trial. ITT will be used in an attempt to avoid potential bias and identify the true effect of the intervention.

The research will use a pre-test-post-test control group approach [33]. This is where pre-intervention (baseline) measures on the prevalence of pregnancy will be conducted to allow for explicit evaluation of the pre-to-post changes.

Qualitative data will be collected through in-depth interviews of key informants in the community and discussions with groups of adolescent boys, parents, community members and school leadership. Standard guides with open-ended questions will be used for conducting the interviews and discussions. The participants of the interviews and discussions will be purposively selected based on their role in preventing adolescent pregnancy in the community.

### 2.1. Variables

#### 2.1.1. Independent Variable: School-Based Sexual and Reproductive Health Services

(a) Availability of services
(b) Accessibility of services
(c) Perceptions towards services and providers

#### 2.1.2. Dependent Variable: Prevention of adolescent pregnancy

Primary outcomes

(a) Incidence of pregnancy
(b) Sexual and Behavior change
(c) Utilization of SRH services

#### 2.1.3. Intervening Factors: Age, Religious Beliefs, Family Background, Level of Formal Education and Socio-Economic Status of the Adolescents.

**Study Area:** This study will be conducted in the Hoima District in Bunyoro region, because it is among the top five regions in Uganda with a high percentage of adolescent pregnancies, and hence, it is a representative region. Hoima has a higher percentage of adolescent pregnancies at 29% compared to the national level of 25% [4]. Access (5 km or more) to a public health facility by the population including adolescent girls is limited and it is estimated that approximately 39.5% of the population in the Hoima District live outside the catchment area (5 km or more) of public health facilities. This contributes significantly to inaccessibility and low utilization of SRH care among the adolescents who largely depend on financial support from the parents or guardians. This implies that the adolescent girls may not have the need for ASRH services met due to this hindrance [4].

The District has a thriving oil and tourism economic activities which increase the risk of vulnerability of the adolescent girls to risky sexual behavior. The Hoima District is located in Western Uganda, approximately 230 kilometers from Kampala. The District has a total geographical area of 5735.3 square kilometers and is bordered by Buliisa, Masindi, Kibaale, Kyankwanzi and Ntoroko Districts, and the Democratic Republic of Congo across Lake Albert. The District has a total of 36 registered secondary schools.

**Study Population:** 1080 adolescent girls aged 15–19 years attending secondary school in the Hoima District, Uganda.

### 2.2. Inclusion Criteria

- Girls aged 15 to 19 completed years at the time of the study
- Currently in secondary school in the Hoima District
- Schools enrolling both sexes or girls-only single sex schools will be included
- Ability to read and write in the English language
- Formal informed consent form signed by the parent or guardian for girls aged 15–17 years
- Formal informed assent form signed by girls aged 18 and 19 years

### 2.3. Exclusion Criteria

- Girls below 15 years or above 19 completed years at the time of study
- Girls who are unable to read and write English language
- Girls with no formal informed consent from parent/guardian and herself

### 2.4. Sample Size

#### 2.4.1. Sample Size Calculation

The sample size will be calculated using the formula for comparing differences in rates and or proportions between two groups and determining the number of subjects per group *^n^*_Individual_Binary_ for a two-sided significance level *α* and power 1 − *β* [31]:

$${}^{n}{}_{\mathrm{Individual}\_\mathrm{Binary}}=\frac{\left({}^{z}{}_{1-\alpha /2}+{}^{z}{}_{1-\beta }(2[{}^{\pi }{}_{T}(1-{}^{\pi }{}_{T})+{}^{\pi }{}_{C}(1-{}^{\pi }{}_{T})\right]}{{\left({}^{\pi }{}_{T}-{}^{\pi }{}_{C}\right)}^{2}}$$

#### 2.4.2. Considerations

*π*T = Proportion of pregnant girls in group 1 approximately 29% (0.29). This is the current pregnancy prevalence in Bunyoro region (29%).

*π*C = Proportion of pregnant girls in group 2 approximately 19% (0.19). This is the expected reduction in prevalence of adolescent pregnancy after the intervention.

$$\begin{array}{lll} \mathrm{Effect}\mathrm{size} & = & 10\%[\delta \mathrm{Binary}=(\pi T-\pi C)],\mathrm{which}\mathrm{is}0.1\\ \alpha & = & 0.05(\mathrm{two}-\mathrm{sided})\\ \beta & = & 0.2\\ Z\alpha /{}_{2} & = & \mathrm{Desired}\mathrm{significance}\mathrm{level}\mathrm{at}5\%(1.96)\\ Z_{1-\beta } & = & \mathrm{Desired}\mathrm{power}\mathrm{at}80\%(0.842)\\ \mathrm{Type}\mathrm{of}\mathrm{test} & = & \mathrm{Two}-\mathrm{sided} \end{array}$$

Absolute risk reduction = 10%;

**Step One:** Formula for determining the number of subjects per group *^n^*_Individual_Binary_ for a two-sided significance level *α* and power 1 − *β*:

$$\begin{array}{c} =\frac{{\left(1.96+0.842\right)}^{2}[0.29(1-0.29)+0.19(1-0.19)]}{{\left(0.29-0.19\right)}^{2}}\\ =(2.802)2[0.2059+0.1539]\\ =(7.851\times 0.3598)/0.01\\ =282.5\\ \sim 283\mathrm{individuals} \end{array}$$

**Step Two:** In order to adjust for the clustering effect, the sample size (*^n^*_Individual_Binary_) was inflated by a Design Effect (DE) to get an adjusted sample size (*^n^*_Cluster_Binary)_ [31]. Therefore, to determine the adjusted sample size per study group (*^n^*Cluster_Binary) for a Cluster Randomized Controlled Trial (cRCT), with a binary outcome, we multiply it with the Design Effect (DE):

*^n^*_Cluster_Binary_ = *^n^*_Individual_Binary_ × Design effect

*^n^*_Cluster_Binary_ = *^n^*_Individual_Binary_ × [1 + (*m* − 1) *ρ*]

where *m* is the fixed cluster size and *ρ* is the Intra-cluster correlation coefficient (ICC). ICC is the ratio of the between-cluster variance to the total variance of an outcome variance and quantifies the correlation between the outcomes of any two individuals within the same cluster [31]. Since there is no previously published data/study in Uganda documenting the ICC, an ICC of 0.01 was considered because it is within the recommended inter-quantile range ICC (0.011–0.094) for previously undocumented ICC. Campbell and Walters [24] recommended a minimum cluster size of 50 subjects per cluster to cater for secondary outcomes; therefore, a fixed cluster size of 60 individuals was considered for this study.

*^n^*_Cluster_Binary_ = *^n^*_Individual_Binary_ × [1+ (*m* − 1) *ρ*] = 282.5 × [1 + (60 − 1) × 0.01] = 449 adolescent girls per group = 449 × 2 = 898 individuals for the entire study (449 per group)

The final total sample size (898) will be increased by 10% to 988 participants to cater for a non-response insurance factor of 10%. Therefore, the treatment and control group will each have 494 adolescent girls.

### 2.5. Number of Clusters

The minimum number of units to be able to achieve a 5% level of significance is four per arm assuming normality for the cluster level responses and the use of non-parametric test, Mann–Whitney *U*-test [24]. Campbell [31] further states that with three units per arm, it is within the bounds of chance that the outcomes in the three treatment arms are all greater than the outcomes in the three control arms; however, with four per arm, this is unlikely to happen by chance more than 1 in 20 times. Therefore, the minimum number of clusters is four per arm (eight in total) for an unmatched design or six matched pairs for a matched design.

**To determine number of clusters needed (*n*_cluster):**

*n*_cluster = Total number of participants in the two groups (/fixed cluster size) = (*^n^*_Cluster_Binary × 2)/*m*_ = 988/60 = 16.5 ~17 clusters.

In order to have the same number of clusters per arm, the number of clusters was increased to the nearest upper even number, which is 18 in this study. Therefore, the study will have a total of 18 clusters; nine clusters will be randomized to the intervention and nine clusters will be randomized to control group. This means that the sample size contains 1080 adolescent girls in 18 clusters.

### 2.6. Sampling Techniques

The study will use multi-stage sampling technique to select the study population. The Hoima District was purposively selected because of the high (29%) adolescent pregnancy and the thriving economic activities, which further increase the vulnerability of the adolescent girls to pregnant. The District has 36 registered secondary schools in four constituencies Kigorobya, Bugahya, the Hoima municipality and the Buhaguzi counties.

**Cluster Sampling:** Clusters of adolescent girls attending secondary school who represent the target population will be identified and included in the sample [34]. This will be used to increase the level of efficiency of sampling and each cluster will be considered as a sampling unit. In this study, the clusters will be secondary schools registered and operational in the Hoima District; a list of all clusters (schools) will be generated from the District Education Office and clusters will be ranked according to the level of school dropout rate. The top 18 clusters (schools) with the highest dropout rate will be considered for the study. The selection will be based on the judgment of the researcher using drop-out rates at each school within the population in the Hoima District. The schools to be selected from each county should rank highest in dropout rates of adolescent girls in the last 12 months prior to the study. Four to five secondary schools with the highest student enrolment and highest school dropout rates will be selected from each county. The statistics will be derived using the annual school dropout reports from the Hoima District Education Officer (DEO) and the Chief Administrative Officer (CAO). In addition to the school dropout rates, the schools to participate in the study will meet the following requirements:

- Permission to conduct outreach SRH services at school and willingness of the head teacher
- Availability of SRH focal person usually called the ‘senior woman teacher’
- Proximity to a functional public health center (within 5–10 km)
- Access to eligible students
- Geographical location of the school

**Random Sampling:** The allocation of schools (clusters) to either the intervention and control groups will be randomly conducted. After selection of the 18 schools, nine schools will be randomly allocated to the intervention group and the other nine will be allocated to the control group. The names of the 18 selected schools will be placed in a basket and randomly selected and allocated to either group of study.

Blinding will be conducted at the random allocation of the school clusters to either the intervention group or control group. The research assistants, Biostatistician and school administration will be blinded to the study hypotheses.

**Systematic Sampling:** Systematic sampling will be used to select the 1,080 adolescent girls from the 18 schools (clusters). This is a probability sampling method in which sample members from a larger population are selected according to a random starting point and a fixed periodic interval [33]. Upon selection of the 18 schools that are considered to be clusters, 60 eligible girls will be systematically selected from each school using class lists.

**Figure 1:**
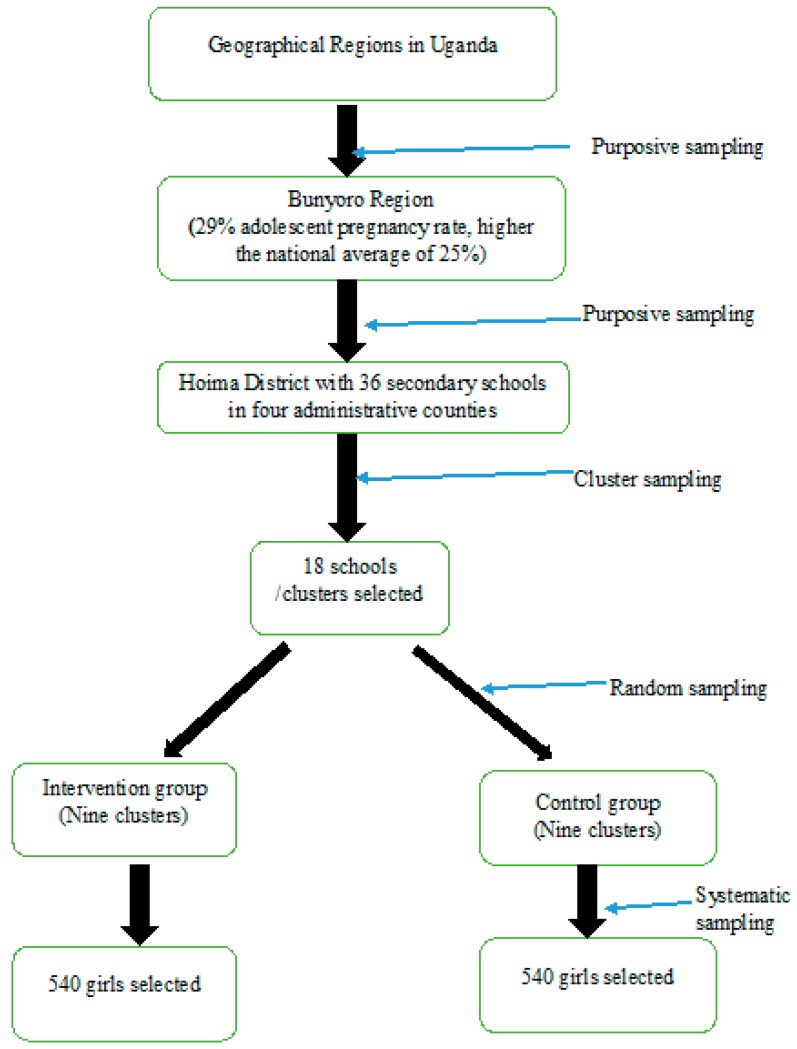
Flow chart on the Sampling framework. Source: Primary data 2018.

A list of all girls aged 15–19 years in form two, three and five will be generated by the school administration. This periodic interval will be determined beforehand as 2, which means that assuming the starting point is 1 as the first serial number on the class lists, every girl with an even serial number will be selected to participate in the study, that is, 2, 4, 6, 8, etc. The girls will have an equal chance to be selected to participate in the study. A total of 1080 secondary school girls will be systematically selected from the 18 schools in equal numbers of 60 girls aged 15 to 19 years per school. Figure 1 illustrates the sampling framework to be used during the study.

### 2.7. Recruitment of Study Participants

The study will be conducted in the 18 selected secondary schools as clusters; each school will be randomly assigned to the intervention study group or the control study group. Each school cluster will be independent, with no crossover of staff/students and defined as a natural cluster for the purpose of the trial. Each arm of the study will have nine schools (clusters).

The selection of the 18 study schools (clusters) will be conducted as per the criteria described in Section 2.6 (sampling) and each of the 18 schools will be randomly assigned to either the intervention (with SBSRH package) or the control group (without SBSRH package). This means that both the intervention arm and control arm had will have a total of nine schools each.

Within each school, the 1080 study participants will be selected systematically based on the inclusion criteria described in Section 2.2 (selection criteria). The recruitment of the study participants will be done independently by the researchers and research assistants. This will be done at school using the systematic sampling framework described in Section 2.6. The process is anticipated to take place within one month for all the 18 schools. Only girls who meet the inclusion criteria will be enrolled into the study. The participants will be informed about the study objectives without revealing the study hypotheses. Upon recruitment, each participant with their parent will be required to ascent and consent, respectively, by signing formal ascent and consent forms. Data collection (base line) will be done, an SBSRH package will be provided in the nine intervention clusters (schools) and follow-up is done for 12 months. An end of study assessment will be conducted after twelve months and data will be analyzed.

### 2.8. Description of the Intervention

The intervention for the experimental group will be a three tier adolescent tailored SRH service package provided at school. The School-Based Sexual and Reproductive Health (SBSRH) package will be provided for 12 months and will include provision of ASRH information and awareness sessions using stepping stones methodology, in-school ASRH services and referral to public health facilities. Follow-up will be done at 6 months and 12 months from the start of the study.

#### 2.8.1. Tier 1: In-School ASRH Education Session

The health education sessions will provide information and create awareness on HIV, pregnancy, safer sex, Gender Based Violence, HIV, pregnancy, sexually transmitted infections (STIs), gender norms, effective communication and safe sex practices, sexuality and self-assertion. The Stepping Stones curriculum and the National sexuality framework in Uganda will be used as the standardized behavioral change package using the interpersonal peer to peer approach. 10 sessions (communication, how we act, sex, love, gender based violence, contraceptives, HIV prevention, safer sex practices, assertiveness, supporting one another and self-reflection) will be conducted for the participants by fellow trained peers and knowledge change as well as behavioral change assessed. Implementation of sessions will be done in small groups of 15–20 girls (peers). Peer facilitators will be trained and supported to conduct the small group discussions on SRH and HIV prevention. The researcher, research assistants, SRH nurse and senior woman teacher in-charge of SRH will supervise these sessions.

#### 2.8.2. Tier Two: In-School Adolescent Sexual and Reproductive Health (ASRH) Services

ASRH services will be provided at the intervention school (on-site) in an allocated room/school clinic. They will be available and accessible to all students regardless of whether they are participating in the study or not. Key services will include information, sensitive counseling, STI screening and treatment, HIV testing, contraceptives (where permitted), referral and general medical care. These will be conducted once a week by a skilled health worker recommended by the health unit in-charge at the school premises from 7:00 am to 6:00 pm. The SBHCs will be operational only during the school terms; however, their operation during the holiday will be dependent on the need and authorization from the school administration.

#### 2.8.3. Tier Three: Referrals to off-Site Public Health Facilities

The students identified in need of advanced ASRH care such as post abortion care, antenatal care and HIV treatment will be referred to primary or tertiary public health facilities for further management. Details of the reasons for referral will be recorded and a Ministry of Health (MOH) referral form filled and given to the student. The health workers at the public health facilities nearest to the school will be sensitized about the study and possible referrals to be made and a mechanism of management of the referred cases will be developed to prevent delays and stigma associated with adolescent SRH care. The district ASRH officer will take lead in this activity for ownership but also to monitor the response of health workers during the implementation.

**Follow-up:** Follow-up of the participants will be done for 12 months and data will be collected at the start of the study, after six completed months and at the end of the study after 12 months. Data on student demographics, services received and perception towards the service and provider will collected on each clinic day to assess the level of utilization and the most sought after services.

**Control Group**: The clusters in this arm will not receive any intervention from the research team. However, the participants are free to continue accessing services from the nearby facilities as has been the case prior to the study.

**Data Collection Methods:** Quantitative data will be collected using the survey method three times that is at the start of the study (0 months), six months and at the end of the study after 12 months. Qualitative data will be collected once during the 12 months of the study through interviews and discussions with the identified stakeholders. This is intended to provide subjective and relevant information to enrich the quantitative data on adolescent pregnancy in the Hoima District.

**Data Collection Tools:** Quantitative data will be collected using a self-administered questionnaire while Qualitative data will be collected using the Key Informant Interview guide and discussions using the Focus Group Discussion guides. Additional qualitative data will be collected at other SRH service delivery point to provide a snapshot on the level of utilization of services provided; this will be done using an SRH utilization register.

**Participants:** The respondents to the questionnaire, key informant interview and focus group discussions will be categorized as shown in Table 1.

**Validation Procedure:** The questionnaire to be used in this study will undergo three types of validity, which include; (1) content validity; (2) criterion- related validity; and (3) construct validity. This validation procedure will ensure that the questionnaire accurately measures what it aims to do, regardless of the responder. A valid questionnaire will help to collect better quality data with high comparability, which reduces the effort and increases the credibility of data.

## 3. Analysis

Analysis will be done using aggregate cluster-level analysis; regression analysis using individual patient data with robust standard errors; random-effects (Res) models using individual patient data; and population averaged models using individual patient data with regression coefficients estimated using generalized estimating equations (GEEs). In this study, data exploration will be done to visualize the general feature of the data then the key analysis will be by SPSS version 20 at both cluster and individual levels. Both descriptive and inferential statistics will be analyzed allowing for the association between SBSRH service and prevention of adolescent pregnancy (occurrence of pregnancy, sexual behavioral change). The intent to treat analysis will be used to analyze patients according to their random treatment assignment, which is the intended treatment, not the treatment actually received.

Descriptive summary statistics of the clusters and individuals such as frequencies, mean, medians and standard deviations will be used to describe the characteristics of the study population on sex, age, socio-economic, education level, pregnancy status and sexual behavior, among others. Summary statistics for categorical data will be displayed in form of mean, proportion, frequencies and percentages depending on the nature of the data.

To determine the association between the dependent and independent variables, a general logistic regression model will be employed and two steps will be followed. First, each variable will be entered into a binary logistic regression model. Second, variables that are found to be significant at a p-value of 0.05 will be fitted into a multiple logistic regression model to identify independent factors of influencing occurrence of adolescent pregnancy. Variables that remain significant at a p-value of 0.05 will be further analyzed using multivariate logistic regression model and variables will be identified as independent factors influencing incidence of pregnancy and behavioral change.

Based on the above logistical regressions, inferential statistics such as t-test and chi square test will be used to determine the similarity in the two groups using their baseline data (baseline comparison) and to determine relationships as well as associations between variables. Chi square tests will be used to test the relationships between categorical variables, while independent t-tests will be carried out to determine differences between the study groups for continuous variables with normal distribution.

Cluster level: The summary measure for each cluster (mean or proportion) will be calculated, because each cluster provides only one data point and the data will be considered to be independent while allowing standard statistical tests to be used. Multiple regressions will be used for the data summarized at the cluster level.

Individual level: During the two stage data analysis process, individual level covariates will be incorporated into the cluster analysis while accounting for the intra-cluster correlation, thus increasing the statistical power of the analysis. Statistical tests will be conducted taking into account for the clustering effect.

Qualitative data collected during the key informant interviews and focus group discussions will be transcribed and typed into a Microsoft Word computer program. These notes will be transcribed verbatim to ensure complete capturing of the responses of the study participants. Thematic analysis will be used to analyze qualitative data from key informant interviews and focus group discussions using the Atlas qualitative data analysis software version 7 (atlasti.com). Themes and sub-themes relevant to the objectives of the study will then be identified to enable development of qualitative coding and code definitions. Codes will be grouped into categories and then themes. The coded transcripts will be analyzed by running query reports and primary documents tables of codes by objective or theme and results will be used to explore the magnitude of the issues from the various focus group discussions and key informant interviews. Quotes illustrating meaning or key messages from the analysis will be selected based on the code count or how illustrative to the theme the quotes will be.

Structural equation modeling (SEM) using Analysis of a Moment Structures (AMOS) and Statistical Package for Social Sciences (SPSS) will be used to determine the statistical significance of the SBSRH model in the prevention of adolescent pregnancy. The constructs of the SBSRH model will be specified based on the theories that informed the research and the findings. Data of the identified constructs will be entered into the SEM software package. The package will fit the data to the SBSRH model and produces the results, which will include the overall model fit statistics and parameter estimates. Matrices of correlations and means will be used and relationships will be expressed as restrictions on the total set of possible relationships. The results feature overall indexes of model fit as well as parameter estimates, standard errors, and test statistics for each free parameter in the model.

## 4. Expected Results

A reduction in the incidence of pregnancy among the participants, behavioral change, utilization of services and increased awareness on sexual and reproductive health.

## 5. Discussion

This study will address central gaps in the evidence base in regard to the prevention of adolescent pregnancy among the school girls. To our knowledge, this is the first CRCT to be conducted in Uganda focusing on adolescent girls in schools and employing combination intervention using the three tire approach. The results of the study will effectively inform programming.

## Figures and Tables

**Table 1:** Categorization of study respondents. ASRH: Adolescent Sexual and Reproductive Health.

	Category of Respondent	Sample Size	Data Collection Tool
1	Adolescent girls (15–19 years) in school	1080	Questionnaire
2	Adolescent girl (15–19 years) who dropped out of school due to pregnancy related reasons	2	Key Informant Interview guide
3	ASRH Health workers	1	Key Informant Interview guide
4	Local council leader (chairperson)	1	Key Informant Interview guide
5	Community leaders	10	Focus Group Discussion guide
6	District leaders (Chief Administrative Officer, District Health Officer, District Education Officer)	3	Key Informant Interview guide
7	School heads	1	Key Informant Interview guide
8	School Matron	1	Key Informant Interview guide
9	Parents to adolescent girls in school	15	Focus Group Discussion guide
10	Adolescent boys (15–19 years) in school	15	Focus Group Discussion guide
11	Teachers	10	Focus Group Discussion guide
12	Domestic workers/axillary staff at school	10	Focus Group Discussion guide

Source: Primary data, 2018.
